# Intelligent deep learning model for targeted cancer drug delivery

**DOI:** 10.1038/s41598-025-96149-6

**Published:** 2025-05-30

**Authors:** Islam R. Kamal, Saied M. Abd El-atty, S. F. El-Zoghdy, Randa F. Soliman

**Affiliations:** 1 Department of Computer Science, Faculty of Information Systems and Computer Science, October 6 University, Giza, 12585 Egypt; 2 Department of Electronics and Electrical Communications Engineering, Faculty of Electronic Engineering, Menoufia University, Menouf, 32952 Egypt; 3 Department of Mathematics and Computer Science, Faculty of Science, Menoufia University, Shebin El-Kom, 32511 Egypt; 4 Machine Intelligence Department, Faculty of Artificial Intelligence, Menoufia University, Shebin El-Kom, 32511 Egypt

**Keywords:** Internet of bio-nano-things, Health care systems, Nano-sensors, Molecular communication, Nano-devices, Nanotechnology, Cancer, Health care, Medical research, Molecular medicine, Engineering

## Abstract

Nanotechnology and information communication technology (ICT) are being combined to develop innovative drug delivery systems for targeted sites, such as tumor cells. The particulate targeted drug delivery (PTDD) system involves drugs containing nanoparticles embedded in nanoscale devices (referred to as bio-nanomachines) that can cross vascular barriers, resulting in an increased concentration of the drug in the targeted cell or tumor. An artificial intelligence bio-cyber interface (AIBCI) operates in both forward and reverse directions, enabling the transfer or control of a desired drug dose without affecting healthy cells, facilitated by the Internet of Biological Nano Things (IoBNT). This paper proposes a multi-compartmental model with an AI bio-cyber interface based on molecular communication technology. The proposed model is formulated as a set of multi-differential equations designed to identify molecular communication-based bio-nanomachines, enabling the quantification of drug concentration at the targeted cell. Unlike conventional compartmental models, the present model is designed to connect both the exterior and interior of the human body. The results suggest that the model has the potential to improve the capacity of target cells to respond to therapeutic drugs while reducing adverse effects on healthy cells. The intra-body nanonetwork proposed in the present study proved superior performance in magnetifying the drug concentrations in diseased cells.

## Introduction

Nanotechnology introduces new concepts and devices that have the potential to enhance existing healthcare technologies while also paving the way for groundbreaking scientific advancements^1^. Based on the idea of manipulating materials at the molecular and nanoparticle scales, nanotechnology enables the development of small, bio-compatible devices (bio-nanomachines) capable of interacting with biological materials at even the organism level. According to^2^, bio-nanomachines are multifunctional devices that can detect, capture, store, release, and combine biological molecules. They can also facilitate locomotion, manipulate objects, and even reproduce or terminate biological processes. Doxorubicin (Dox) is an anticancer medication used to treat various tumors; however, its dosage is limited due to risk of cardiotoxicity. This has led researchers to explore optimal dosing regimens that magnify the drug’s anti-tumor effects while minimizing the risk of cardiotoxicity linked to plasma concentration^3^. Nanotechnology enables the development of nanoparticles (NPs) that can deliver and release drugs directly at tumor site. Additionally, nanotechnology suggests that MC can be used to interconnect these bio-nanomachines. MC is significant because of its ability to enhance understanding of complex diseases, such as cancer, by analyzing fundamental intracellular and extracellular processes within interacting bio-nanomachines. This system can also be managed comprehensively using externally controlled mechanisms. The Internet of Biological Nano-Things (IoBNT)^4–6^, based on AI bio-cyber interfaces, presents a promising framework for connecting the MC environment with external networks, such as the Internet.

Molecular Communication has made a significant contribution to nanomedicine, particularly in the emerging field of targeted drug delivery systems (TDDS), which is gaining considerable attention^7,8^. The primary goal of TDDS is to send a specific drug to the required site (the targeted area) while preventing the drug from negatively affecting healthy cells^9^. In a TDDS scenario based on MC, a transmitter nanomachine transports drug particle via drug molecules, which serve as information carriers. The blood plasma network acts as the communication channel, while the targeted or tumor tissue functions as the receiver nanomachine^7,10^.

This paper proposes a multi-compartmental framework for a TDDS based on IoBNT to regulate therapeutic drug concentration through an AI bio-cyber interface model. The proposed framework enhances drug concentration in tumor tissues, leading to reduced therapeutic doses, improved efficacy, minimized toxicity, and better patient compliance and convenience. It establishes two-way communication between medical personnel and bio-nanomachines embedded in the human intra-body network. Additionally, the AI bio-cyber interface is designed for IoBNT, facilitating interaction with the external environment to control and adjust drug particle concentrations within targeted cells. The cyber interface employs deep learning to identify model parameters, with a trained network capable of predicting system behavior. The deep learning-based technique is used to estimate the design parameters of AI bio-interface model in IoBNT, similar to a TDDS. Root Mean Square Error (RMSE) and processing time are compared between non-linear least squares curve fitting and deep learning-based approaches. The AI bio-interface enables the translation of various detected molecular signals, facilitating communication between diverse micro-networks within the human body.

## Related work

Incorporating health monitoring through implantable medical devices and information and communication technology (ICT) via the IoBNT represents the future of healthcare technology. A framework for IoBNT, highlighting a scenario where it enhances healthcare delivery was presented in^11^. The framework includes a bio-cyber interface that connects a biochemical signaling-based bio-nano-network to the electromagnetic-based internet. One of the main challenges for IoBNT is ensuring effective drug delivery to targeted cells, which is not always guaranteed by traditional methods. To address this issue, the model was updated with new parameters to improve drug delivery. The focus of the analysis is on system’s bio-cyber interface and the blood vessel information diffusion network, which leads to intra-body nano-network.

In^9^, a TDD based on MC is introduced, sending therapies to infection sites for achieving TDD effectiveness was investigated. In this study, nano-transmitters and nano-receivers are injected into the specified blood network, and drug molecules are delivered to targeted sites using a multi-compartmental model. Drug delivery efficacy is achieved by targeting the surface of tissues. However, the effectiveness of the process depends on various factors such as receiver size, diffusion characteristics, the volume capacity of nano-transmitters, and enzyme concentration. Despite these advancements, the actual control and delivery of drug particles to intended sites, as well as the eradication process, were not addressed by the IoBNT model in this work.

The study in^12^ describe a new TDDS paradigm and present improvements in doxorubicin delivery systems that enhance therapeutic efficacy and safety. This study also reviews existing FDA-approved doxorubicin formulations but does not employ the IoBNT framework for remote control and targeted delivery of drug particles. A mathematical model explaining the cellular uptake, cytotoxicity of doxorubicin, and the chemotherapy drug as used to treat cancer was introduced in^3^. This model assumes that the cell survival rate is dependent on drug-induced damage, following a sigmoidal, and Hill-type pattern. The study’s findings reveal that doxorubicin’s cytotoxicity is influenced by both intracellular and extracellular mechanisms.

Recent advancements in IoBNT and its potential to improve TDD, particularly in oncology, were explored in^13^. In^14^, the authors employed a bioinspired MC system where molecules are used as information carriers, and a multi-input multi-output (MIMO) Förster resonance energy transfer (FRET) nanorelay is utilized to efficiently transfer therapeutic drugs from extravascular (outside blood vessels) to intravascular (within blood vessels) sites targeting diseased tissue. A comprehensive review of the technical aspects of IoBNT and MC, focusing on their potential for drug delivery and biomedical monitoring, is provided in^15^. The application of IoBNT in healthcare, emphasizing its role in precise medical interventions such as drug delivery, is thoroughly described in^16^. Furthermore, a novel bio-nano-thing (BNT) architecture, called Molecular Nano Neural Networks (M3N), was introduced in^17^. This structure allows for the implementation of intelligence at the micro-/nano-scale and consists of compartments (low complexity entities) that are connected to form a network.

In the present manuscript, we enhance the biochemical model by capturing the intricate dynamics of the tumor microenvironment, including the tumor’s extracellular matrix, intracellular pathways, and biochemical reactions involved in drug interactions. To achieve this, we incorporate a multi-compartmental model that considers both the macroscopic behavior of drug delivery and the biochemical interactions at the tumor site. These modifications provide a more realistic and detailed representation of tumor-drug interactions. Additionally, our proposed model incorporates tumor microenvironmental factors, including the extracellular matrix (ECM), pH levels, and oxygenation, all of which impact the drug’s bioavailability and effectiveness. Moreover, our model integrates drug kinetics and pharmacodynamics by including a pharmacokinetic/pharmacodynamic (PK/PD) model to simulate drug distribution and interaction within the tumor compartment more accurately. This accounts for factors such as drug absorption, distribution, metabolism, and excretion, which is providing a clearer understanding of how drug concentrations evolve over time. Further, the study addresses tumor-specific biochemical reactions by introducing reactions that reflect how different tumor types may respond to specific drugs^17–20^.

Previous studies highlighted therapeutic drug delivery to unhealthy cells in specific areas, but, to the best of our knowledge, none employed IoBNT to monitor and control nano-carrier diffusion within the body area network (BAN). Thus, our proposed framework aims to deliver therapeutic medications while controlling drug particle concentration at the target site to minimize undesired effects on healthy cells in the same region. As a result, this study presents a TDDS based on MC and a multi-compartmental model that employs an AI bio-cyber interface within the IoBNT paradigm to regulate the drug delivery process. Additionally, the computational impact of system design specifications related to IoBNT configurations is also investigated.

## Proposed system model

### System model descriptions

**Fig. 1:**
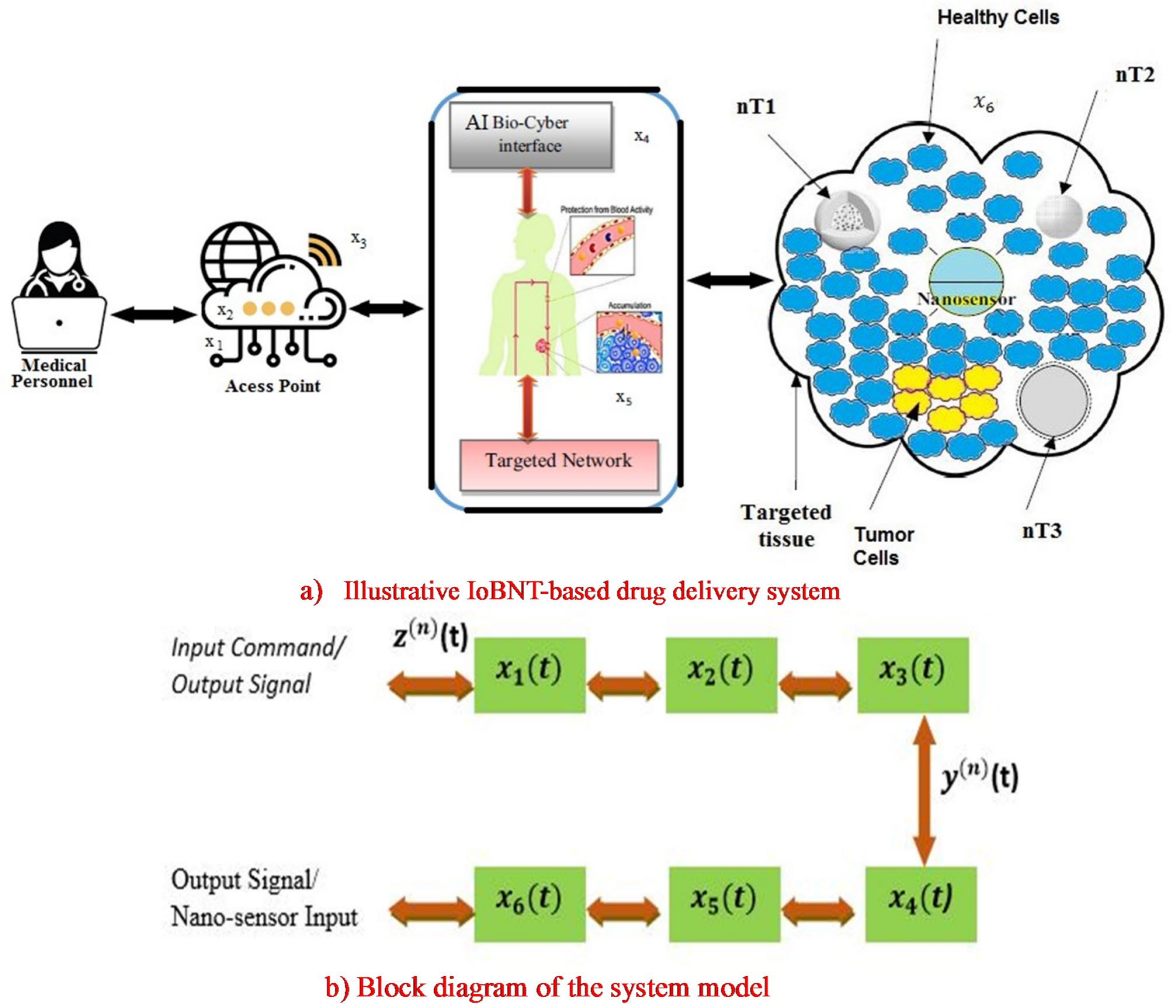
Proposed system model descriptions.

In the present work, the IoBNT model described in^21^ is modified and further developed, as illustrated in Fig. 1. The scenario involves a TDDS where the drug DOX is administered to specific cells in the body. The nanonetwork consists of therapeutic nanomachines (n$\:{T}_{1}$, n$\:{T}_{2}$, n$\:{T}_{3}$, and a bio-nano-sensor) placed within intra-body environment. A smart injection pump, as described in^22^, regulates injection of DOX drug-loaded nanomachines. The transport of DOX drugs is facilitated through a MC system^8,10^. The bio-nano-sensor monitors the targeted nanonetwork to detect chemical signals. Inspired by biological cell structures, therapeutic nanomachines are equipped with receptors that bind to specific ligands (drugs), facilitating the reception process through a ligand-receptor binding mechanism. Furthermore, these therapeutic nanomachines can transmit and receive data, functioning as transceiver nano-devices. Assume that n$\:{T}_{1}$ is transceiver bio-nanomachine (similar to a PEGylated liposome) that encloses injected drug. In this process, n$\:{T}_{1}$ can receive stimuli signals to release the DOX drug. Nanomachines n$\:{T}_{2}\:$and n$\:{T}_{3}\:$support the system by synthesizing and releasing specific particles that enhance the drug delivery process.

The present model enhances the IoBNT framework by incorporating a two-way bio-cyber communication interface to enable precise, real-time drug delivery to tumor tissue. It explores how nanoparticles (DOX drugs) are exchanged among different bio-nanomachines. In the forward direction, the received binary command activates a combinational logic circuit that generates a thermal or light response, stimulating n$\:{T}_{1}$ (PEG liposome) to release DOX drugs. In the reverse direction, the bio-nano-sensor detects biochemical signals in the blood network and converts them into electrical signals. The bio-nano-sensor identifies information particles in the blood vessel generated by a bioluminescence reaction, and the bio-cyber interface transmits this signal to the patient via the Internet. Thus, the primary function of the bio-cyber interface is to connect the external network (such as the Internet) with the targeted nanonetwork (tissue).

As illustrated in Fig. 1(a), signals $\:{x}_{1}$, $\:{x}_{2}$, $\:{x}_{3}$, $\:{x}_{4}$, $\:{x}_{5}$ and $\:{x}_{6}\:$facilitate the communication sequence in both forward and reverse directions. The primary objective of the proposed IoBNT-based drug delivery system is to assist medical personnel in the healthcare delivery process. Medical personnel act as the remote managers responsible for storing, managing, and maintaining information related to the regulation of the DOX drug delivery system. To send specific instructions, the remote medical personnel utilize a wireless portal (access point) that connects the AI bio-cyber interface to both the remote personnel and the patient’s body via the Internet.

The proposed model employs cloud-based settings and beacons to enable knowledge exchange among doctors and central system to manage device interactions, and mobile wireless stethoscope devices used by physicians. The trained data supports the IoBNT system within the cloud environment, and the central system, as illustrated in Fig. 1, ensuring DL training. Following this, the Amboyer interface transmits the signal to the designated group of therapeutic bio-nanomachines within the target tissue via a complex vascular network. The therapeutic bio-nanomachines are designed to detect malignant sites without harming the surrounding healthy tissues.

As shown in Fig. 1(a), the AI bio-cyber interface collects data from the bio-nano sensor and converts it into an electrical signal in the reverse direction. In the forward direction, the bio-cyber interface translates traditional electromagnetic waves into biochemical signals, which trigger a specific bio-nanomachine, such as nT_1_ (e.g., PEGylated liposomes), to release stored nanoparticles (DOX drug). These nanoparticles diffuse through the bloodstream and pass-through defective blood vessels that supply the tumor.

The block diagram of the proposed system model is shown in Fig. 1(b). Inspired by conventional wireless communication systems, the modules $\:{x}_{1}$(*t*), $\:{x}_{2}$(*t*), and $\:{x}_{3}$(*t*) represent the conventional IoT system, while module $\:{x}_{6}$(*t*) represents a group of bio-nanomachines and the bio-nano sensor in the targeted nanonetwork. The focus of this study is the physical layer structure of the proposed system model, represented by $\:{x}_{3}$(*t*), $\:{x}_{4}$(*t*), and $\:{x}_{5}$(*t*). The effects of losses and errors in $\:{x}_{1}$(*t*) and $\:{x}_{2}$(*t*) caused by data transmission from medical personnel at the access point have been ignored. Consequently, the communication channel is designed using $\:{x}_{1}$(*t*), $\:{x}_{2}$(*t*), and $\:{x}_{3}$(*t*) with the output of the wireless communication channel $\:{z}^{n}$(*t*) as follows:

1 $$\:{z}^{n}\left(t\right)={y}^{\left(n\right)}\left(t\right)*\left({x}_{1}\left(t\right)*{x}_{2}\left(t\right)*{x}_{3}\left(t\right)\right),\:n=\{f,\:r\}$$

Where $\:{\:y}^{\left(n\right)}$is the input signal, and the convolution operator is denoted by *. The variables *n*,* r* and *f* represent the communication direction type, communication direction, and reverse communication direction, respectively.

### System model analysis

**Fig. 2:**
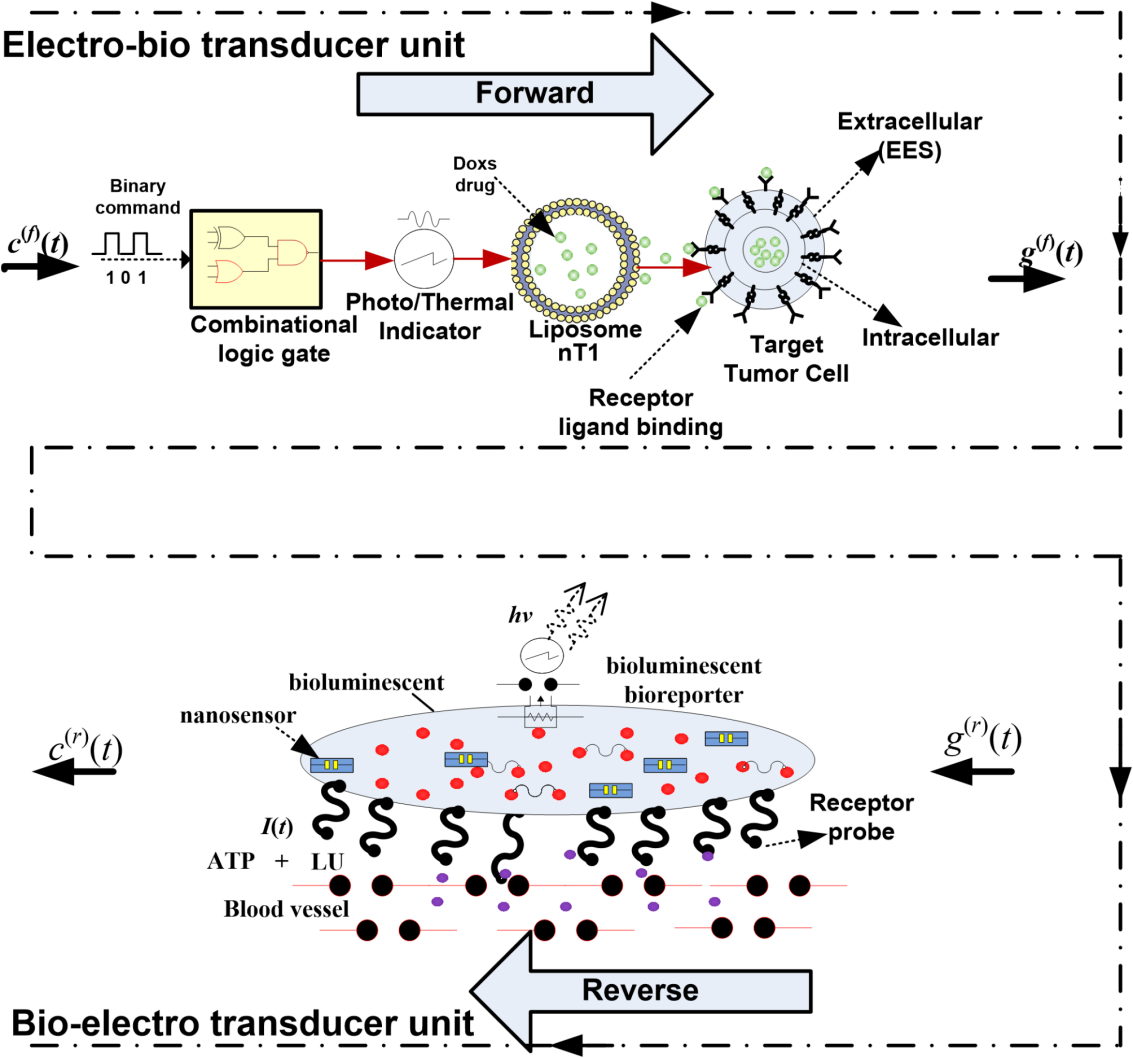
The developed dual bio-cyber interface model.

The proposed system model primarily relies on the architecture of bio-cyber interface. Consequently, we developed an interface that includes a dual-transducing unit to decrypt a command message executed by an electromagnetic (EM) wave and then utilize that command to operate a transducer unit for generating a biochemical signal, as illustrated in Fig. 2. The developed interface depends on $\:{c}^{\left(f\right)}$(*t*) and $\:{c}^{\left(r\right)}$(*t*), which represent the electrical and biological signals in both directions. The concept of developed bio-cyber interface, which converts electrical signals to biochemical signals and vice versa using a combination of logic gate circuits from^11^, is employed in the present paper.

**Fig. 3:**
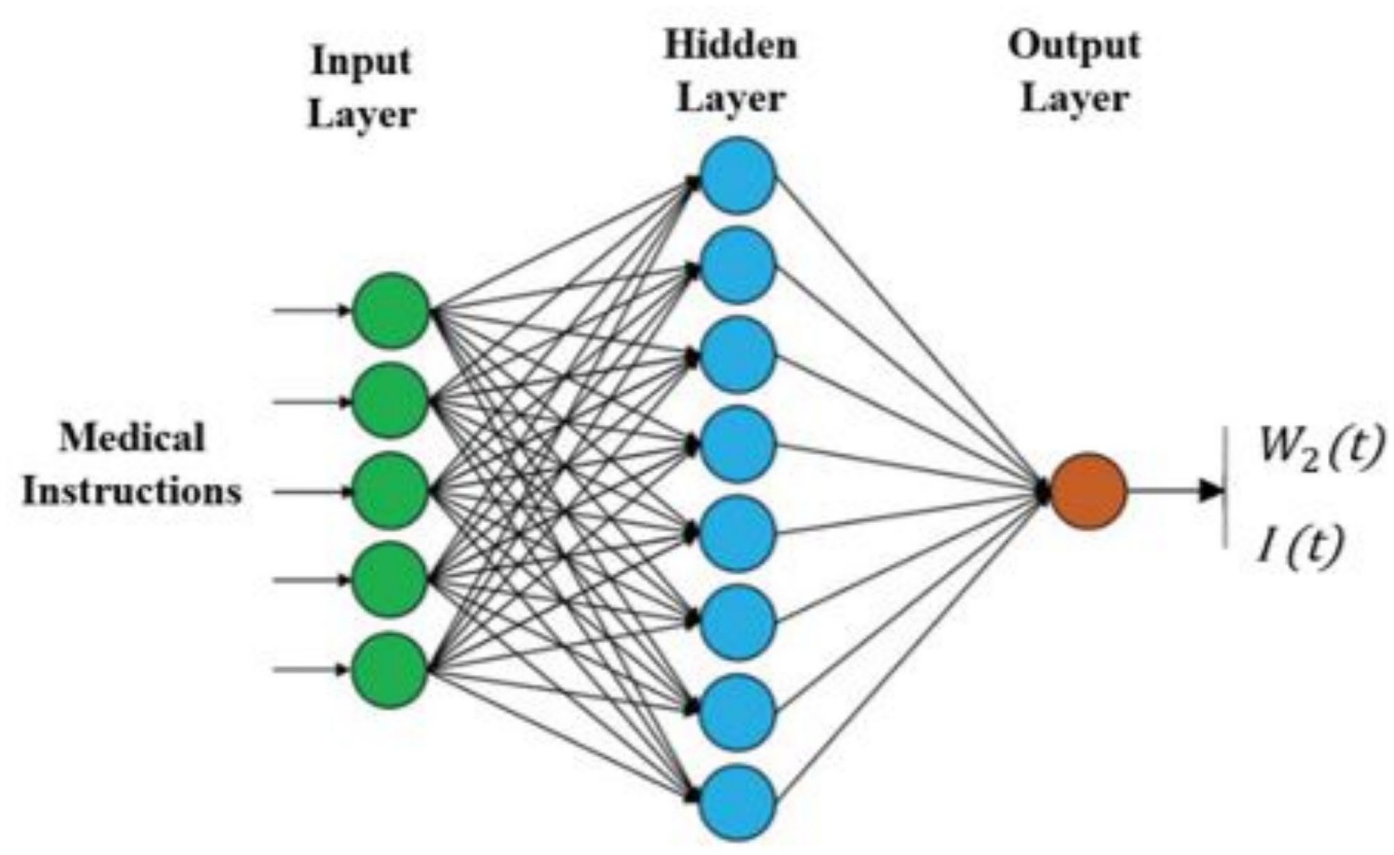
Artificial Neural Network (ANN) model diagram.

The functions of the proposed bio-interface technique are carried out through a series of steps. First, an Artificial Neural Network (ANN) is applied to the dataset obtained from fitting to learn the model parameter design; Fig. 3 illustrates the ANN model diagram^23–25^. Second, a nonlinear least squares technique is used to fit the parameters of the models. During the simulation, M observations are made using nonlinear least squares fitting.

The model parameters estimation is expressed as Eq. (2).

2 $$\:Fitting\left\{\begin{array}{c}argmin{\sum\:}_{i=1}^{M}\begin{array}{c}({W}_{2}\left({t}_{i},{S}_{1},\dots\:\dots\:.,{S}_{n}\right)-{V}_{1}\left({t}_{1}\right){)}^{2}\\\:{S}_{1},\dots\:,{S}_{n}\end{array}\\\:argmin{\sum\:}_{i=1}^{M}\begin{array}{c}(I\left({t}_{i},{S}_{1},\dots\:\dots\:.,{S}_{n}\right)-{V}_{2}\left({t}_{1}\right){)}^{2}\\\:{S}_{1},\dots\:,{S}_{n}\end{array}\end{array}\right.$$

The model fitting parameters $\:{S}_{1}$, …, $\:{S}_{n}$ account for fitting simulation data corresponding to results of the electro-bio and bio-electro units up to time *t*, as denoted by $\:{V}_{1}\left({t}_{1}\right)$ and $\:{V}_{2}\left({t}_{1}\right)$, respectively. These parameters are utilized to minimize the evaluation function. The parameters ($\:{s}_{1},{s}_{2},{s}_{3},{s}_{4}$) comprise the output of the nonlinear least-squares fitting technique. ANN is a group of interconnected nodes used for recognizing and resolving modeling issues that have complex causal relationships and solutions. A multilayer perceptron neural network (MLPNN) is employed for prediction and nonlinear processes. The use of ANN in this context provides a significant nonlinear structural function. In Eq. (3), a value of 10 is assigned to the number of hidden layers while the MLPNN is applied to learn and predict the model parameters. Essentially, the nodes of the hidden layer must be connected to all input layer nodes, and at the same time, all the hidden layer nodes are connected to every node in the output layer. The weights *W* and activation function *f* utilize a log-sigmoid function that scales data values between 0 and 1. Furthermore, before applying the ANN, the feature data values are scaled to a range of 0 to 1, as shown in Eq. (3).

3 $$\:Function\left\{\begin{array}{c}f:y\in\:{\mathbb{R}}^{D}⟶z\in\:{\mathbb{R}}^{1}\\\:f\left(y\right)={b}_{2}+{\omega\:}_{2}*\left({f}_{A}\left({b}_{1}+{\omega\:}_{1}*y\right)\right)\end{array}\right.$$

4 $$\:{y}^{*}=\frac{y-{y}_{min}}{{y}_{max}-{y}_{min}}$$

Where $\:{y}^{*}$ represents the input features after training, and $\:{y}_{min}\:\text{a}\text{n}\text{d}\:\:{y}_{\text{m}\text{a}\text{x}}\:$are the minimum and maximum input feature values, respectively. $\:{z}_{k}$ is calculated as shown in Eq. (5).

5 $$\:\left\{\begin{array}{c}z=f(W\:{y}^{*}+b)\\\:{z}_{k}\left(y,\omega\:\right)={f}_{1}({b}_{k}+{\sum\:}_{i=1}^{m}{\omega\:}_{kj}{f}_{2}({b}_{j}+\sum\:_{i=1}^{n}{\omega\:}_{ji}{y}_{n}^{*}\left)\right)\end{array}\right.$$

The activation functions utilized in the ANN are denoted by $\:{f}_{1}$ and $\:{f}_{2}$, while *n*,* m*, and *k* represent the input nodes, hidden nodes, and output nodes, respectively. The weight from the input unit $\:{\:x}_{i}$ to the hidden unit $\:{y}_{i}\:$is given by $\:{w}_{ji}$, and the weight from the hidden unit $\:{y}_{i}$ to the output unit $\:{z}_{k}$ is represented by $\:{w}_{kj}$. The biases for the hidden and output layers are denoted by $\:{b}_{j}$ and $\:{b}_{k}$, respectively. To reduce undesired measurement effects, the ANN introduces noise errors. Bayesian regularization combines the square errors and weights to simplify the model without overfitting. The trained ANN is then utilized to estimate the bio-interface parameters for different scenarios^26–28^.

In the forward direction, the received binary command can use a combinational logic circuit to produce a thermal or photo response that stimulates nT_1_ (PEG Liposome) to release the DOX. In MC technology, the DOX drug, as nanoparticles, is stored in nT_1_ (thermal/photo sensitive). The electro-bio unit $\:{g}^{\left(f\right)}$(*t*) in the forward direction is given by:

6 $$\:{g}^{\left(f\right)}={\int\:}_{0}^{{R}_{IN}}\xi\:\omega\:\left(t\right)dt,\:{\omega\:}_{0}={\left.\xi\:\omega\:\left(t\right)\right|}_{t={R}_{IN}}$$

*R*_*IN*_ denotes the time difference between *t*_*A*_ and *t*_*R*_, while $\:\xi\:$ represents total number of liposomes. $\:\omega\:\left(t\right)\:$denotes DOX concentration released at the specific time t. $\:{\omega\:}_{0\:}$is the value of $\:{g}^{\left(f\right)\:}$that is required the targeted site. The IoBNT system is employed to manage period *R*_*IN*_ and $\:{{\upomega\:}}_{0}$ to obtain the optimal DOX concentration.

In the proposed model, the nT_1_ encapsulated DOX drugs are injected intravenously, passively propagate through the blood vessel network, and enter the targeted site. nT_1_ acts as the stimulator at time *t*, and probability density function of DOX released by nT_1_ is as follows^29^:

7 $$\:\omega\:\left(t\right)={d}_{t}{\omega\:}_{R}\left(1-{e}^{-\delta\:t}\right)$$

$\:{{\upomega\:}}_{R}$ represents the emitted DOX concentration with a rate of δ, equivalent to the first-rate constant. Based on the stimulation pattern, there are two release rates for temperature and light, represented as *δ*_*t*_ and *δ*_*l*_, respectively, with both *δ*_*t*_, and ***δ***_*l*_ being subsets *δ.*

In Eq. (2),$\:\:{d}_{t}=\frac{d}{dt}$ and $\:{{\upomega\:}}_{\text{R}\:}$≈$\:\underset{0}{\overset{\infty\:}{\int\:}}{\upomega\:}\left(t\right)dt$.

In the reverse direction, biochemical signals detected in the blood network are converted to electrical output by the bio-nanosensor. Due to insufficient drug delivery, malfunction, or other factors, the bio-nanosensor detects variations in molecular data from targeted cell and delivers them as proteins to the necessary part. The bio-cyber interface receives molecular information released by the bio-nanosensor, converting it into an equivalent electrical signal. Additionally, this molecular information can activate cellular pathways through receptor interaction with intravascular probes or direct diffusion, influenced by computed transcription factors, as depicted in Fig. 2. The bio-luminescent reaction described in^11^ generates a phosphate group (PP), adenosine monophosphate (AMP), and light (hv). The intensity of the bio-luminescence I(t), represented by Eq. (8), is influenced by luciferase (LU) and adenosine triphosphate (ATP) and it can be modeled using the Michaelis-Menten mechanism^11^.

8 $$\:I\left(t\right)=\frac{{\alpha\:}_{L}LX}{X+{\alpha\:}_{m}}$$

In Eq. (8), *X* and *L* are the concentrations of ATP and LU, respectively. $\:{\:\alpha\:}_{m}$, $\:{\alpha\:}_{L}$ represent the Michaelis-Menten and catalytic reaction constants, respectively. L can be computed by utilizing the differential equations for mRNA gene expression^30^, along with the Hill function specified in^31^. According to the proposed ligand-receptor interaction in^32^, the concentration of diffusing information molecules determines the activation of the transcription factor within the cellular structure. This activation can occur through either receptor-mediated or direct diffusion mechanisms, contrasting with the statement declared in^33^. The concentration of the transcription factor, which reflects the proposed bio-cyber interface, is depicted in Eq. (9).

9 $$\:m\left(t\right)\approx\:\mu\:{g}^{\left(r\right)}$$

where$\:\:{g}^{\left(r\right)}\left(t\right)$ represents the reverse direction biological signals and *µ* is the cellular structure surface signal conditioning.

## The proposed framework based on a molecular communication

**Fig. 4:**
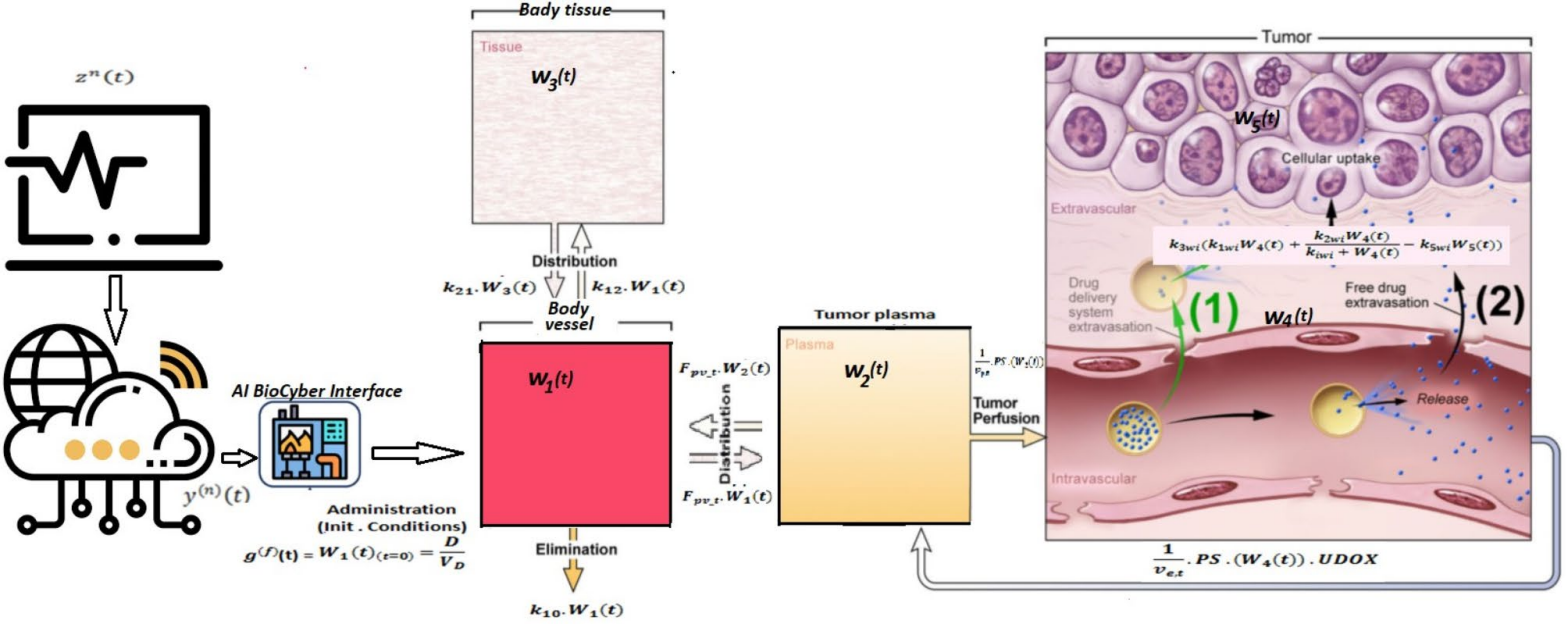
Flow diagram of proposed multi-compartmental framework.

Figure 4 illustrates the MC flow in the blood vessel (or cardiovascular) network utilizing our proposed framework, where *w*_1_(*t*) and *v*_1_(*t*) represent the concentrations of DOX molecules in both directions. The model focuses on accurately delivering a dose of DOX to the tumor cell. A bio-cyber system is integrated to certify the model’s effectiveness, enabling seamless operation between the nanonetwork in the human body and the Internet domain. The delivery of DOX from tumor plasma to tumor tissue utilizes ODE-PDE models based on pharmacokinetic principles. Extracellular drugs diffuse within the tumor cord, while absorption and efflux by tumor cells follow saturable absorption kinetics. Tumor cell mortality is influenced by the maximum concentration of the intracellular dose. Furthermore, the DOX concentration in each compartment is determined by the percentage of molecules within the compartment volume. Therefore, in the subsequent subsection, we present an analysis of MC within the multi-compartment framework, examining both forward and reverse directions. Table 1 provides detailed descriptions of the main used parameters.

**Table 1:** Description of the proposed framework main parameters.

Parameter	Description
$\:{\omega\:}_{0}$	Drug concentration injected
$\:{k}_{12}$	Kinetic constant
$\:{k}_{10}$	Elimination Rate
$\:{k}_{21}$	Constant from tissues to blood
$\:{\text{k}}_{1{\text{w}}_{\text{i}}}$	Parameter for intracellular uptake
$\:{\text{k}}_{2{\text{w}}_{\text{i}}}$	Parameter for intracellular uptake
$\:{\text{k}}_{3{\text{w}}_{\text{i}}}$	Parameter for intracellular uptake
$\:{\text{k}}_{5{\text{w}}_{\text{i}}}$	Parameter for intracellular uptake
$\:{\text{k}}_{\text{i}{\text{w}}_{\text{i}}}$	Parameter for intracellular uptake
$\:\text{U}\text{D}\text{O}\text{X}$	DOX Plasma binding
$\:\text{p}\text{s}$	DOX Permeability surface area product
Fpv_t	Plasma flow in tumor plasma space; note *F*pv = blood perfusion (W0) / blood volume (BV)
rho_t	Density for liver tissue
$\:{w}_{1}$	Initial concentration in Blood vessel
D	Total does of encapsulated DOX injected
VD	Distribution volume
$\:{w}_{3}$	concentration in body Tissue
$\:{w}_{2}$	Tumor plasma concentration
$\:{w}_{4}$	Tumor tissue concentration
$\:{w}_{5}$	Concentration in tumor cell
Vp_t	Fractional volume of tumor plasma spaces
Ve_t	Fractional volume of tumor extracellular space
Vv_t	Fractional volume of tumor vascular spaces
Hct_t	Hematocrit for tumor microvasculature
$\:\text{U}\text{D}\text{O}{\text{X}}_{\text{e}}$	Binding for DOX to proteins in EES
Vt_t	Volume fraction of tumor EES

### The forward direction

The forward path from the bio-cyber interface to the targeted nanonetwork involves developing the concentration of the DOX drug through the following steps:

- Step 1: Remote medical personnel send a binary command signal through the network.
- Step 2: An AI bio-cyber interface processes incoming signals and transmits them to an electro-bio transduction unit, which converts electromagnetic waves into corresponding bio-signals. As illustrated in diagram 3, the electro-bio transduction unit decodes signals came from the access point and activates a set of logic gates, producing either a thermal or optical effect. These effects stimulate release of specific molecules encapsulated within nanocarriers. Certain nanocarriers, such as liposomes, can be engineered to release their contents in response to environmental triggers like alterations in temperature, light intensity, pH levels, magnetic fields, or the presence of enzymes^10^. In the proposed design, two sets of liposomes are utilized: one responsive to temperature changes and the other to light variations. Each set is loaded with distinct molecules. As depicted in Fig. 2, when the decoded signal corresponds to the binary code 011, thermal source adjacent to the liposome storage unit is activated, triggering the release of enzymes from thermally responsive liposomes. Similarly, when decoded signal is binary code 111, optical source is activated, stimulating photo-responsive liposomes to release their contents. The released molecules diffuse through a designated region (VR) into the injection chamber, where they are automatically administered into the bloodstream utilizing a painless injection technique. The findings in^22^ demonstrate that painless injection can be achieved by using pressure, vibration, or temperature modulation (cooling/warming) to the skin. Once injected, the molecules traverse circulatory system and passively reach targeted site, where they activate intended nanodevices.
- Step 3: The DOX drug is injected into the bloodstream via the injection machine in the bio-cyber interface, where DOX molecules are gathered and sent to tumor plasma. Passive and active targeting mechanisms ensure that treated molecules remain targeted at the required cells, evading the body’s immune system.
- Step 4: The tumor plasma compartment delivers the drug to the tumor tissues extracellular space (EES). DOX drugs passively diffuse according to permeability-surface area product, taking the varying volumes of tumor plasma compartment and EES, along with drug transport between the tumor plasma and systemic plasma compartments through blood perfusion.
- Step 5: This study assumes two distinct mechanisms for the intracellular uptake of doxorubicin: (1) passive diffusion through the cell membrane and (2) active transport involving reception by nano-transceivers in the tumor tissue EES and subsequent uptake by tumor-targeted cells.

**Fig. 5:**
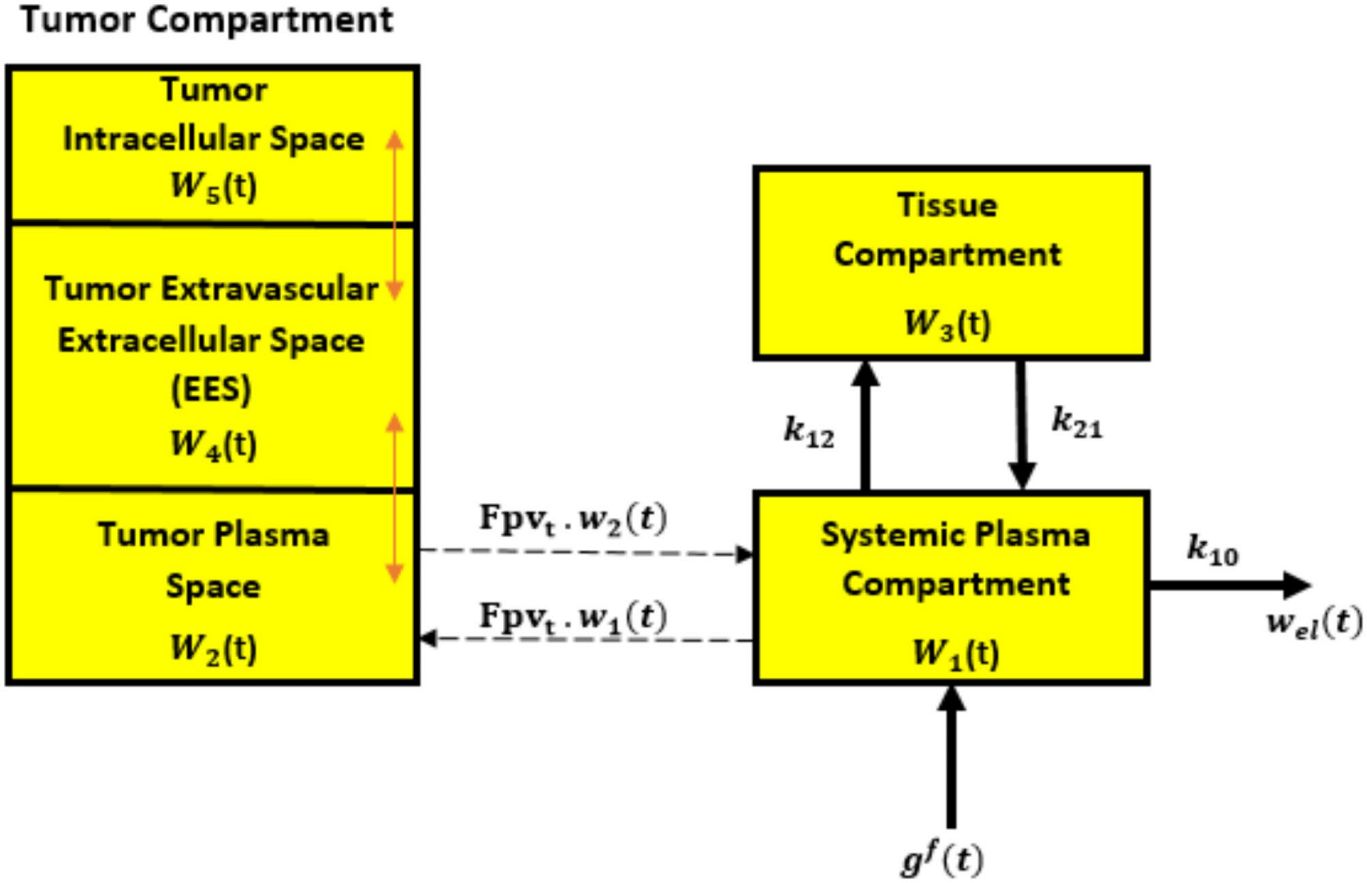
Molecular compartmental model in forward path.

The model shown in Fig. 5 utilizes differential equations obtained from the forward compartment model as follows:

10 $$\:{d}_{t}{w}_{1}\left(t\right)=-{w}_{1}\left(t\right)\left({k}_{12}+{k}_{10}\right)+{k}_{21}{w}_{3}\left(t\right)$$

11 $$\:{d}_{t}{w}_{3}\left(t\right)={k}_{12}{w}_{1}\left(t\right)-{k}_{21}{w}_{3}\left(t\right)$$

The subscripts 0, 1, and 2 denote the positions of the compartments. With initial conditions $\:{w}_{1}$(0) = $\:{g}^{\left(f\right)}$ and $\:{w}_{2}$(0) = 0, the rate at which the medicine concentration changes in the tumor plasma compartment is defined as:

12 $$\:{d}_{t}{\text{w}}_{2}\left(\text{t}\right)\:\:\:\:=-\frac{1}{\text{v}{\text{p}}_{\text{t}}}\times\:\text{p}\text{s}\left({\text{w}}_{2}\left(\text{t}\right)\times\:\:\text{U}\text{D}\text{O}\text{X}\right)+\frac{1}{\text{v}{\text{p}}_{\text{t}}}\times\:\text{p}\text{s}\left({\text{w}}_{4}\left(\text{t}\right)\times\:\text{U}\text{D}\text{O}{\text{X}}_{\text{e}}\right)-\text{F}\text{p}{\text{v}}_{\text{t}}\times\:{\text{w}}_{2\:}\left(\text{t}\right)+\:\text{F}\text{p}{\text{v}}_{\text{t}}\times\:{\text{w}}_{1\:}\left(\text{t}\right)$$

The first term signifies transvacuolar transport via passive diffusion, depending on the permeability surface area product ps and accounting for volume variations between the tumor plasma compartment and the EES. The subsequent expressions demonstrate the amounts of DOX drug transferred through blood perfusion among the plasma tumor and systemic plasma compartments.

Conversely, the change in doxorubicin concentration within the tumor tissue EES is expressed by the following rate equation:

13 $$\begin{aligned} d_{t} {\text{w}}_{4} \left( {\text{t}} \right) = & - \frac{1}{{{\text{ve}}_{{\text{t}}} }} \times {\text{ps}}\left( {{\text{w}}_{2} \left( {\text{t}} \right) \times {\text{UDOX}}} \right) - \frac{1}{{{\text{ve}}_{{\text{t}}} }} \times {\text{ps}}\left( {{\text{w}}_{4} \left( {\text{t}} \right) \times {\text{UDOX}}_{{\text{e}}} } \right) \\ & - {\text{k}}_{{3{\text{w}}_{{\text{i}}} }} \times \left( {\frac{{{\text{k}}_{{1{\text{w}}_{{\text{i}}} }} \times {\text{w}}_{{4{\text{~}}}} \left( {\text{t}} \right) \times {\text{~UDOX}}_{{\text{e}}} + {\text{~k}}_{{2{\text{w}}_{{\text{i}}} }} \times {\text{w}}_{{4{\text{~}}}} \left( {\text{t}} \right) \times {\text{~UDOX}}_{{\text{e}}} }}{{\left( {{\text{k}}_{{{\text{iw}}_{{\text{i}}} }} + {\text{w}}_{{4{\text{~}}}} \left( {\text{t}} \right) \times {\text{~UDOX}}_{{{\text{e~~}}}} } \right)}} - {\text{k}}_{{5{\text{w}}_{{\text{i}}} }} \times {\text{w}}_{5} \left( {\text{t}} \right)} \right) \\ \end{aligned}$$

Here, UDOX represents the plasma binding of the DOX drug, while $\:{\text{U}\text{D}\text{O}\text{X}}_{e}$denotes the binding of DOX proteins in the EES. The first term in Eq. (13) expresses the transvacuolar transmission of the DOX drug between the tumor plasma compartment and the EES. The expressions below detail the transmission of drug from the tumor EES into the tumor cells, while the tumor cell drug concentration is represented in Eq. (14):

14 $$\:{d}_{t}{\text{w}}_{5}\left(\text{t}\right)\:\:\:\:={\text{k}}_{3{\text{w}}_{\text{i}}}\times\:\left({\text{k}}_{1{\text{w}}_{\text{i}}}\times\:{\text{w}}_{4}\left(\text{t}\right)+\frac{{\text{k}}_{2{\text{w}}_{\text{i}}}\times\:{\text{w}}_{4}\left(\text{t}\right)}{{(\text{k}}_{\text{i}{\text{w}}_{\text{i}}}+{\text{w}}_{4}\left(\text{t}\right))\:}-{\text{k}}_{5{\text{w}}_{\text{i}}}\times\:{\text{w}}_{5}\left(\text{t}\right)\right)$$

### The reverse direction

**Fig. 6:**
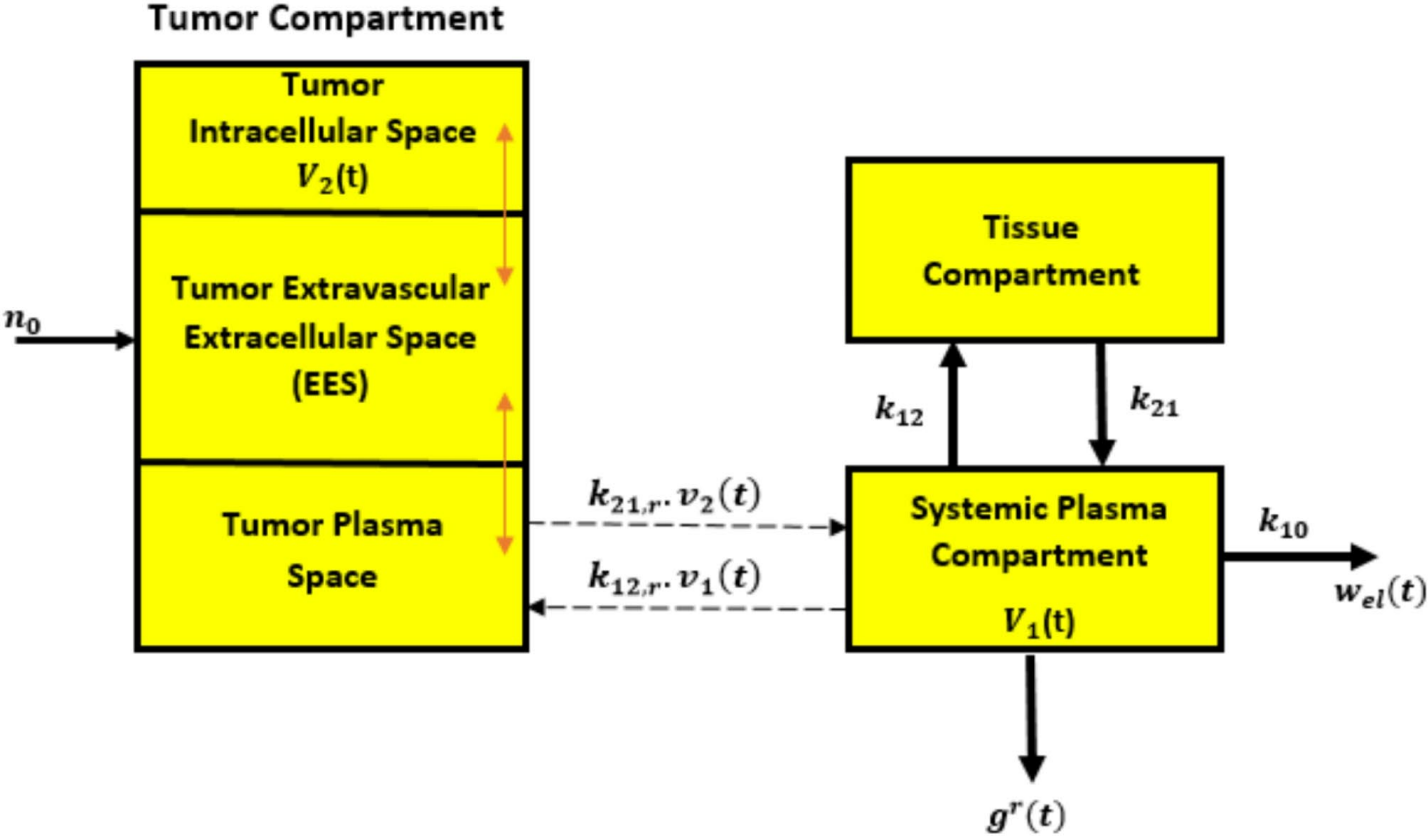
Molecular compartmental model in reverse path.

In this path, depicted in Fig. 6, the systemic plasma represents the central compartment. In this compartment, $\:{v}_{1}$*(t)* is the concentration of molecules, while $\:{v}_{2}$*(t)* represents the concentration of particles in the tumor intracellular space. This concentration encompasses molecules that undergo phagocytosis, react, adhere, and may be absorbed through non-targeted tissues, while concurrently being eliminated by the liver, as discussed in^11^. The parameters $\:{k}_{12,r}$ represent the first-rate constants for the influx and efflux of the targeted nano-network compartments. The rate constants involved in these processes are influenced by various factors, such as the concentration gradient between compartments, the size of the fenestra in the endothelial cell network, and the properties of the diffusing information molecules, as described in^34^. The traditional set of equations used for the multi-compartment model is as follows:

15 $$\:{g}^{\left(r\right)}\left(t\right)={k}_{1}{v}_{1}\left(t\right)$$

16 $$\:{d}_{t}{v}_{1}\left(t\right)={k}_{21,r}{v}_{2}\left(t\right)-\left({k}_{12,r}+{k}_{10}+{k}_{1}\right){v}_{1}\left(t\right)$$

17 $$\:{d}_{t}{v}_{2}\left(t\right)={k}_{12,r}{v}_{1}\left(t\right)-{k}_{21,r}{v}_{2}\left(t\right)$$

$\:{k}_{1\:}$is the ligand-receptor binding constant and $\:{k}_{12,r}$ and $\:{k}_{21,r}\:$are the kinetic rate constants.

The values *t* = 0, $\:{v}_{1}$(0) = 0 and $\:{v}_{2}$(0) =$\:{n}_{0}$ are considered as initial conditions. Additionally, $\:{n}_{0\:}$represents total molecules concentration produced by the bio-nanosensor. Based on the expected value of *I*(*t*), the different transitional conditions of $\:{n}_{0}$ are estimated using IoBNT technology, which is introduced by the bio-cyber interface to $\:{c}^{\left(r\right)}\left(t\right)$. The value of $\:{c}^{\left(r\right)}\left(t\right)$ is either 1 for *I*(*t*) ≥ $\:{I}_{o}$, or 0 for *I*(*t*) <$\:{I}_{o}$, based on Eq. (18).

18 $$\:{c}^{\left(r\right)}\left(t\right)=\left\{\begin{array}{c}1\:\:\:\:\:I\left(t\right)\ge\:{I}_{0}\\\:0\:\:\:\:\:I\left(t\right)<{I}_{0}\end{array}\right.$$

## Simulation results and discussions

In this section, the performance of proposed multi-compartmental model with IoBNT-based bio-cyber interface is evaluated. The evaluation of proposed approach considers the delivery of doxorubicin drug particles to the targeted tumor cells while minimizing side effects on healthy cells and ensuring the responsiveness of these cells before binding to the drug. The effectiveness of the framework in both forward and reverse directions is assessed based on the efficacy of parameters such as *ps*,$\:\:{k}_{3{\text{w}}_{\text{i}}},{k}_{12}$,$\:\:{k}_{21}$and $\:{k}_{10}$, as well as the varying values of certain factors in the forward and backward paths. We execute the simulation using Python to validate our study results. The standard parameters depicted in Table 1 are employed in every calculation. We also used previous experiments conducted in^11^ to select the implemented parameters for each scenario.

**Table 2:** Default values for simulation parameters.

Parameters	Values
$\:{\omega\:}_{0}$	0.7^11^
$\:{k}_{12}$	9.4e-3
$\:{k}_{10}$	2.1e-3
$\:{k}_{21}$	7.052e-5
$\:{\text{k}}_{1{\text{w}}_{\text{i}}}$	2.257^3^
$\:{\text{k}}_{2{\text{w}}_{\text{i}}}$	0.0452^3^
$\:{\text{k}}_{3{\text{w}}_{\text{i}}}$	2.806e-4^3^
$\:{\text{k}}_{5{\text{w}}_{\text{i}}}$	10^3^
$\:{\text{k}}_{\text{i}{\text{w}}_{\text{i}}}$	5.29e-4^3^
UDOX	1.0
PS	4.9e-3^35^
Fpv_t	W0/BV
BV	6.3/100.0*rho_t
rho_t	1.06 g/ml
$\:{w}_{1}$	D/VD
D	0.7
VD	100
$\:{w}_{3}$	Calculated
$\:{w}_{4}$	Calculated
$\:{w}_{5}$	Calculated
Vp_t	Vv_t* (1-Hct_t)
Ve_t	0.454
Vv_t	0.092^36^
Hct_t	0.19^37^
UDOX_e	1.0
Vt_t	0.454^36^

### Performance evaluation in the reverse direction

In this section, the performance of the normalized bio-luminescence intensity, which provides molecular information to the bio-cyber interface in the reverse path of the proposed framework, is evaluated. To do this, we limit the present study to the development of evaluation expressions used in previous experimental work^11^. The values used in the proposed framework are:$\:\:{k}_{21}$= 7.052e^−5^$\:{\text{m}\text{i}\text{n}}^{-1}$, $\:{k}_{12}$ = 9.4e^−3^$\:{\text{m}\text{i}\text{n}}^{-1}$ and $\:{k}_{10}$= 2.1e^−3^$\:{\:\text{m}\text{i}\text{n}}^{-1}$^38^; $\:{k}_{12,r}$= 0.103$\:\times\:{10}^{-2}{\text{m}\text{i}\text{n}}^{-1}$, $\:{k}_{21,r}$= 0.373 $\:{\text{m}\text{i}\text{n}}^{-1}$; and $\:{k}_{1}$ = 0.1$\:\times\:{10}^{-2}{\text{m}\text{i}\text{n}}^{-1}$. We set $\:\mu\:$ = 1 by considering that DOX drug molecules diffuse promptly into the bio-electro transducer unit. The expressions used for translation and degradation rates of LU can be found as$\:\:{k}_{r}\:=\:0.1\times\:{10}^{2}$, $\:{k}_{p}\:=\:1.5\times\:{10}^{2}\:{\text{h}}^{-1}$^39^, $\:{\gamma\:}_{r}=\:0.1005\times\:{10}^{2}{\text{h}}^{-1}$ and $\:{\gamma\:}_{p}=\:0.0415{\times\:{10}^{2}\text{h}}^{-1}$^40^, respectively. For the bio-luminescence reaction, the factors values are, $\:{\alpha\:}_{M}\:=\:15\:\mu\:\text{M},{\alpha\:}_{l}\:=0.044\:\:$and $\:{a}_{tp}\:=0.04\times\:{10}^{3}\mu\:\text{L}\:$^40^. In the simulation, the noise variance value is taken as 0.5$\:\times\:{10}^{-1}\mu\:\text{M}\:$^41^.

**Fig. 7:**
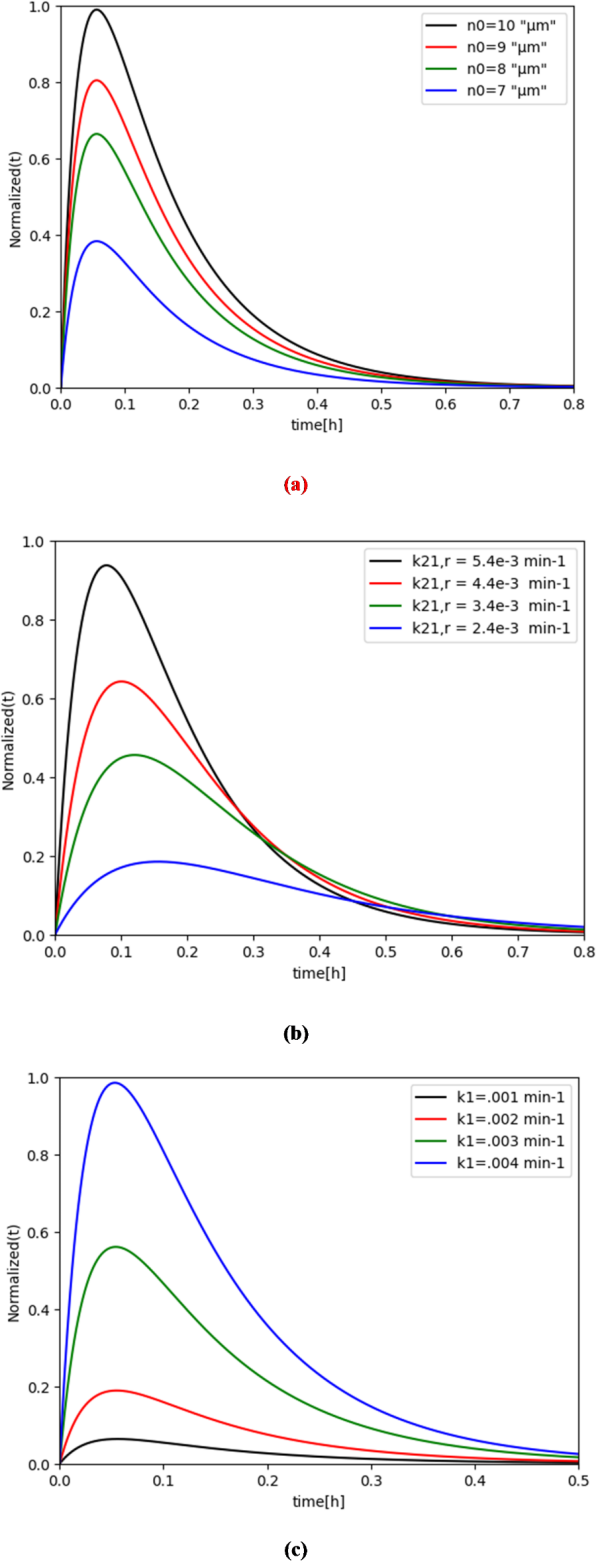
Bio-luminescence density variation with (**a**) concentration of $\:{n}_{0},$ (**b**) the diffusion rate constant$\:\:{k}_{21,r}$, and (**c**) $\:{k}_{1}$.

Additionally, we consider the concentrations of DOX drug molecules as $\:{n}_{i}\left(t\right)\:\text{a}\text{n}\text{d}{\:w}_{i}\left(t\right)\:$ in the presence of Gaussian noise, following a normal distribution with a mean of 0 and a variance of $\:{\sigma\:}_{2}$^42^. To analyze the bio-luminescence intensity, it is assumed that the wavelength of the emitted light remains constant, which is valid if the light beam does not vary significantly. Hence, the sensitivity of the photoresistor in the bio-cyber interface is initially determined by the intensity of the emitted light. The value of$\:{\:\gamma\:}_{l}\:=\:1.04\:{\text{e}}^{-4}$min^−1^ is used in our simulation procedure based on the experimental data in^43^ for liposome exposure to ultraviolet light (UV). To conduct the simulation, the value of $\:{\gamma\:}_{t}$ was set to 0.0078 min^−1^, as determined in previous experiments conducted at a temperature of 42 °C using the non-linear least squares method^44–46^. The effects of varying the values of $\:{n}_{0},{k}_{21,r},{k}_{1},\:{\alpha\:}_{M},{a}_{tp}\:\text{a}\text{n}\text{d}\:{k}_{10}$ on the bio-luminescence density *I*(*t*), described in arbitrary units (a.u.), for accomplishing communication between the bio-cyber interface and medical personnel are shown in Fig. 7.

In the reverse direction, we assume that $\:{I}_{0}$ = 0.007$\:\times\:{10}^{2}$a.u. on the normalized scale. As shown in Fig. 7(a), a low value of $\:{n}_{0}$ (i.e., ≤ 8 µM) is sufficient to deliver the electrical signal in the bio-electro interface, enabling effective acceptance at the bio-cyber interface. Furthermore, it is found that more than one bio-nanosensor is essential, as the bio-nanosensors need to release substantial molecular information to amplify the concentration *n*_0_.

The variation in $\:{k}_{21,r}$ significantly impacts bio-luminescence intensity, as demonstrated in Fig. 7(b). As mentioned earlier regarding the reverse direction, the size of the fenestra in endothelial cells, which controls the exchange between the bloodstream and the targeted site, influences $\:{k}_{21,r}$. Moreover, the diffusion potentials of molecular information and the concentration gradient between compartments $\:{v}_{2}\:$and $\:{v}_{1}$ strongly affect the parameter $\:{k}_{21,r}$. An increase in $\:{k}_{21,r}$ leads to a greater reception of molecular information by the bio-cyber interface, resulting in heightened bio-luminescence, as depicted in Fig. 7(b). The intensity of bio-luminescence shows a marked increase corresponding to the rate at which molecular information is detected by the bio-cyber interface, as shown in Fig. 7(c). This amount is calculated based on probe concentration and the arrangement of porous membranes facilitating the passage of molecular information through the bio-cyber interface.

The accuracy of our proposed model is demonstrated in Fig. 7, which shows that it closely aligns with the numerical solution. Moreover, in Fig. 7(c), it is evident that I(t) increase with rise of $\:{k}_{1}$, and both ANN and non-linear least square curve fitting approaches are in good agreement with the simulation data. This result suggests that ANN is a reliable tool for modeling and predicting IoBNT parameters, and model output can be used for the system parameter analysis and prediction in IoBNT.

### Performance evaluation in forward direction

In the current study, a multi-compartmental model with an AI bio-cyber interface, based on MC technology is proposed. The model is formulated as a set of differential equations used to identify MC-based bio-nanomachines, allowing for the quantification of drug concentration in targeted cells. Unlike common compartmental models, the proposed model is designed to connect both the exterior and interior of the human body.

**Fig. 8:**
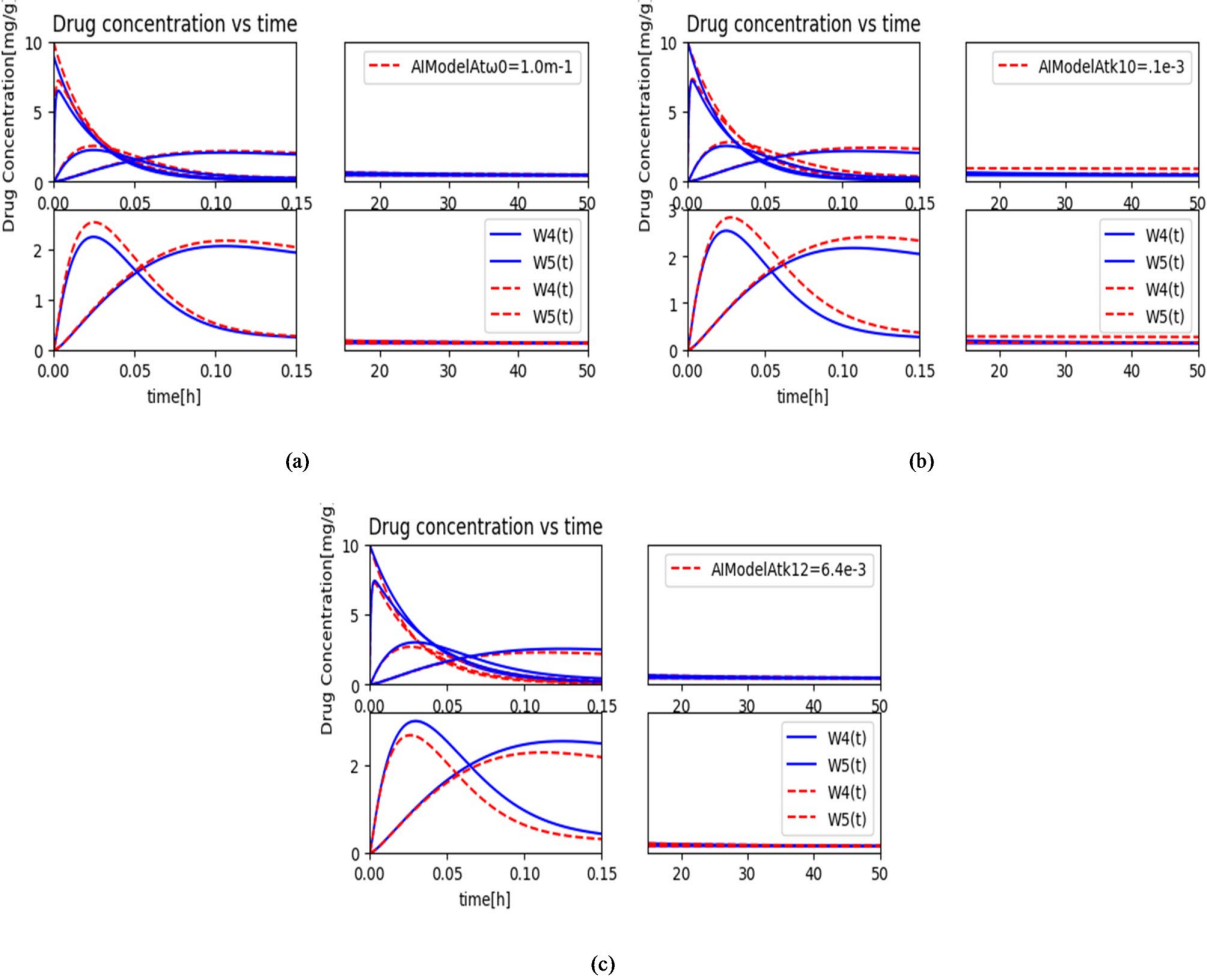
Variation in $\:{w}_{2}\left(t\right),{w}_{4}\left(t\right)\:and\:{w}_{5}\left(t\right)$ sent to the intra-body nano-network with (**a**) $\:{\omega\:}_{0}$, (**b**) $\:{k}_{10}$, and (**c**)$\:\:{k}_{12}$

**Fig. 9:**
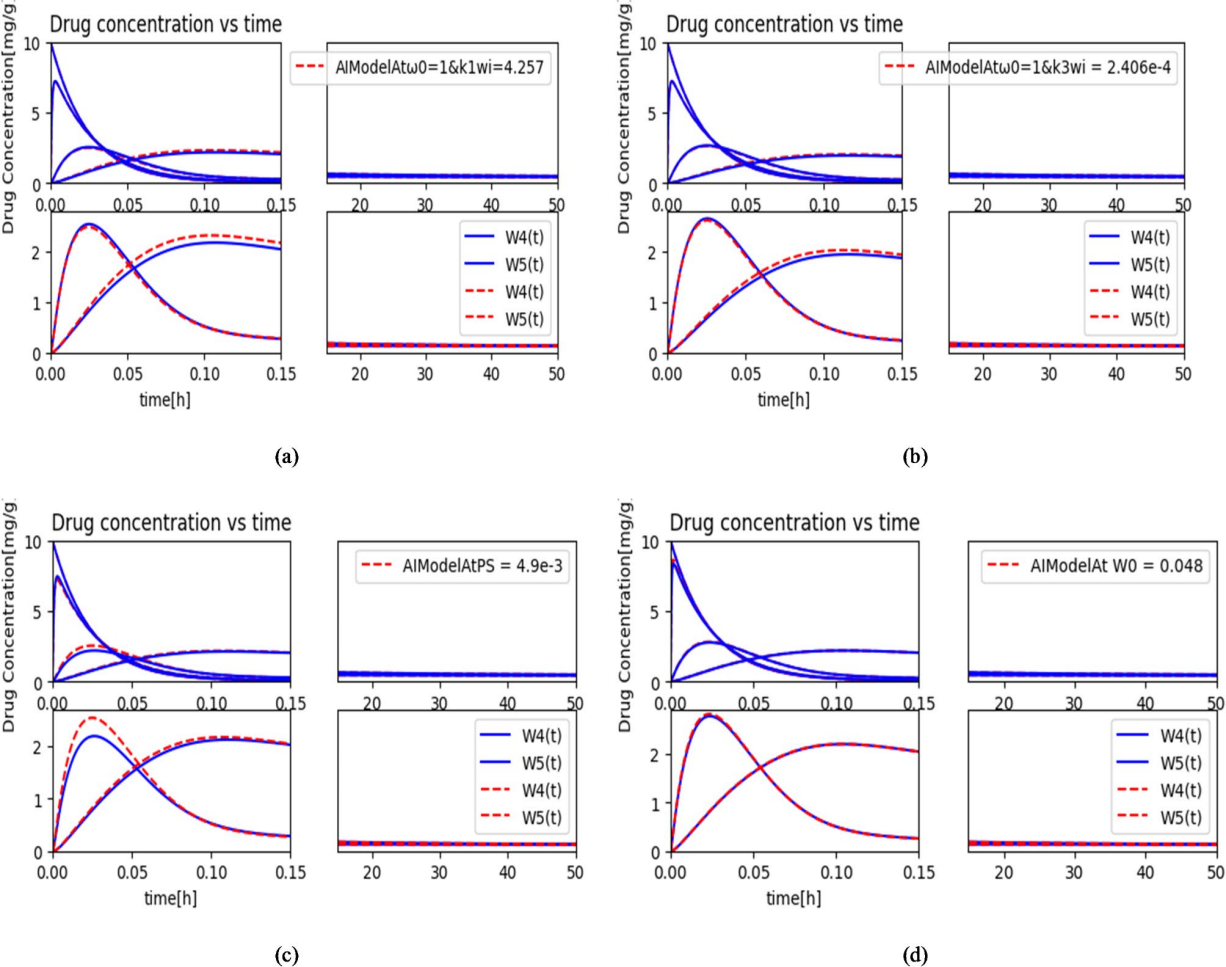
Variation in $\:{w}_{2}\left(t\right),{w}_{4}\left(t\right)\:\text{a}\text{n}\text{d}\:{w}_{5}\left(t\right)$ delivered to the intra-body nano-network with (**a**), and (**b**) Cell uptake parameters and their effect on $\:{w}_{5}\left(t\right)$, (**c**) effect of *ps* on $\:{w}_{5}\left(t\right),\:$(**d**) effect of blood perfusion $\:{w}_{0}$.

The drug concentration bound to tumor cells at various parameters is depicted in Figs. 8 and 9 over the time. To achieve the desired results, we used Eqs. (6) and (10) through (14). The simulation parameters utilized for the present framework are shown in Table 2. The graphs in Figs. 8 and 9 illustrate the effect of different system parameter values on the concentration of molecules over the time. The simulation results demonstrate that the model accurately approximates the numerical solution. In the forward interaction between the AIBCI provider and the sensor network, the most critical parameter is $\:{k}_{10}$. The curves for $\:{w}_{4}\left(t\right)$ and $\:{w}_{5}\left(t\right)$ increase and then decrease after a certain period, as shown in Figs. 8 and 9. Similarly, in the interaction between the sensor network and the AIBCI provider in the IoBNT system, the crucial parameter is $\:{k}_{1\:}$. The ANN proves to be a promising tool for modeling and predicting IoBNT parameters, as evidenced by the close similarity between the ANN-generated curves and those from non-linear least square curve fitting. Furthermore, the trained ANN generalizes the fitted model parameters well, as demonstrated by the graphs in Figs. 8 and 9. Overall, our proposed model effectively validates its capability to predict the IoBNT system parameters.

The fraction of injected $\:{\:\omega\:}_{0}$, which is $\:{g}^{\left(f\right)}$ is controlled by *δ* and $\:{R}_{\text{I}\text{N}}$between the molecule’s release and injection time, as illustrated in Fig. 8(a). Additionally, Fig. 8(a) shows the impact of varying values of the injected molecules $\:{\omega\:}_{0}$, coming from the AI bio-cyber interface enclosed in liposomes and diffusing through the blood vascular network $\:{w}_{1}\left(t\right)$. A higher concentration of emitted molecules increases the drug particle concentration near the reception area of the targeted cell $\:{w}_{5}\left(t\right)$ within the targeted nanonetwork (targeted tissue) $\:{w}_{4}\left(t\right)$. This suggests that before the emitted particles effectively reach nT_3_ as information particles to regulate the dose, they first arrive at nT_2_ as drug molecules, enhancing their interaction with the targeted cells.

In Fig. 8(b), we observe the impact of varying the elimination rate $\:{k}_{10}$ on the results. These illustrations clearly show that $\:{k}_{10}\:$affects both the drug particle concentration in diseased cells, $\:{w}_{5}\left(t\right)$, and, consequently, IoBNT. A higher value of this parameter accelerates elimination, resulting in a reduced peak plasma concentration and a shorter duration of effects. Thus, $\:{w}_{5}\left(t\right)$ is a critical parameter when designing IoBNT systems.

Figure 8(c) illustrates how an increased forward rate constant $\:{\:k}_{10}$augments the drug particle concentration near the reception area within targeted tissues, $\:{w}_{4}\left(t\right)$. It is also evident that this parameter is influenced by compartmental differences, the scale of fenestration connecting the nanonetwork to blood network through endothelial cell networks, and the characteristics of diffusing information particles.

The current model’s evaluation of forward cell uptake parameters $\:{\text{k}}_{1{\text{w}}_{\text{i}}},{\text{k}}_{3{\text{w}}_{\text{i}}}$ is also analyzed using the tabulated values from Table 1. The effects of the forward rate constants $\:{\text{k}}_{1{\text{w}}_{\text{i}}},{\:\text{k}}_{3{\text{w}}_{\text{i}}},\:$*ps*, and $\:Fpv\_t$ are illustrated in Fig. 9(a) to (d), respectively. Additionally, it is noted that $\:Fpv\_t$ depends on blood perfusion $\:{w}_{0}$ and blood volume in liver parenchyma (BV).

Figure 9(a) and (b) illustrate the impact of the forward cell uptake parameters $\:{\text{k}}_{1{\text{w}}_{\text{i}}},{\text{k}}_{3{\text{w}}_{\text{i}}}$ on the tumor cell (targeted cell) concentration *w*_5_(*t*). Without external assistance, cellular uptake of molecules and drug carriers is typically inefficient. Therefore, the effective intracellular delivery of chemical entities requires the development of robust delivery systems. A multi-compartment model influenced by IoBNT has been employed to enhance the system’s efficacy in safely and effectively delivering drugs to targeted cells, as shown in Fig. 9(a) and (b). Increasing $\:{\text{k}}_{1{\text{w}}_{\text{i}}},{\text{k}}_{3{\text{w}}_{\text{i}}}$ visibly enhances the value of *w*_5_(*t*), in the targeted cell, thereby increasing drug concentration within the tumor cell.

Figure 9(c) illustrates the influence of the permeability surface area product, *ps*, on the concentration of drug molecules in targeted tumor cells, *w*_5_(*t*). The *ps* parameter reflects the vascular wall’s ability to facilitate the transmission of small molecules to the targeted area. Understanding the permeability surface area is crucial, as the vascular wall acts as a barrier, preventing larger molecules from reaching tumors. As shown in Fig. 9(c), a higher *ps* value enhances drug flow to the targeted cells, increasing the concentration of drug molecules within infected cells and improving treatment efficacy. Therefore, when designing IoBNT, *ps* is a critical parameter to consider.

The impact of w_0_ on plasma concentration is shown in Fig. 9(d). This parameter plays a significant role in increasing the information concentration transmitted from the bio-cyber interface to bio-nanomachines within the targeted tissue, $\:{w}_{4}$ (*t*), which, in turn, enhances drug delivery to the targeted cell, $\:{w}_{5}$ (*t*). A higher *w*_0_ leads to a higher concentration of molecules in the required cells. In this case, the model demonstrates the effectiveness and impact of *w*_0_ on molecule concentration. As a result, *w*_0_ is also an important parameter in designing IoBNT systems. All the above findings suggest that the model has the potential to enhance the capacity of target cells to respond to therapeutic drugs while minimizing negative impacts on healthy cells.

## Conclusion

This paper analyzes a proposed multi-compartmental model utilizing IoBNT technology, envisioned as a promising approach for enhancing healthcare delivery. The IoBNT model integrates an AI bio-cyber interface, using the ANN technique to estimate bio-interface parameters. The model, formulated as a set of differential equations, is designed to identify molecular communication-based bio-nanomachines and quantify drug concentrations in targeted cells. Unlike traditional compartmental models, this approach connects both the exterior and interior of the human body. Its performance, validated through RMSE and time processing, showed encouraging results for bio-interface modeling. Once trained, the ANN effectively estimates bio-interface parameters, making the IoBNT system a practical and innovative diagnostic tool. The results suggest that the model enhances the ability of targeted cells to respond to therapeutic drugs while reducing harmful effects on healthy cells. Future enhancements aim to develop a more robust decision support system in healthcare, capable of diagnosing various diseases and supporting treatment decisions. Further, the intra-body nanonetwork proposed in this study showed superior performance in increasing drug concentrations in diseased (tumor) cells.

## Data Availability

Data availability The datasets used and/or analysed during the current study available from the corresponding author on reasonable request.
